# hSSB1 (NABP2/ OBFC2B) is required for the repair of 8-oxo-guanine by the hOGG1-mediated base excision repair pathway

**DOI:** 10.1093/nar/gkv790

**Published:** 2015-10-10

**Authors:** Nicolas Paquet, Mark N. Adams, Vincent Leong, Nicholas W. Ashton, Christine Touma, Roland Gamsjaeger, Liza Cubeddu, Sam Beard, Joshua T. Burgess, Emma Bolderson, Ken J. O'Byrne, Derek J. Richard

**Affiliations:** 1 School of Biomedical Research, Institute of Health and Biomedical Innovation at the Translational Research Institute, Queensland University of Technology, Woolloongabba, QLD 4102, Australia; 2 School of Science and Health, University of Western Sydney, Penrith, NSW 2751 Australia; 3 School of Molecular Bioscience, University of Sydney, Sydney, NSW 2006, Australia

## Abstract

The maintenance of genome stability is essential to prevent loss of genetic information and the development of diseases such as cancer. One of the most common forms of damage to the genetic code is the oxidation of DNA by reactive oxygen species (ROS), of which 8-oxo-7,8-dihydro-guanine (8-oxoG) is the most frequent modification. Previous studies have established that human single-stranded DNA-binding protein 1 (hSSB1) is essential for the repair of double-stranded DNA breaks by the process of homologous recombination. Here we show that hSSB1 is also required following oxidative damage. Cells lacking hSSB1 are sensitive to oxidizing agents, have deficient ATM and p53 activation and cannot effectively repair 8-oxoGs. Furthermore, we demonstrate that hSSB1 forms a complex with the human oxo-guanine glycosylase 1 (hOGG1) and is important for hOGG1 localization to the damaged chromatin. *In vitro*, hSSB1 binds directly to DNA containing 8-oxoguanines and enhances hOGG1 activity. These results underpin the crucial role hSSB1 plays as a guardian of the genome.

## INTRODUCTION

Eukaryotic cells are subject to constant DNA damage, with the oxidation of nucleotides being the most common form of damage in unperturbed cells (1). Guanine, due to its chemical structure, is the most frequently modified base and forms 8-oxo-7,8-dihydro-guanine (8-oxoG) after oxidation (1,2). As 8-oxoG may pair with either cytosine or adenine nucleotides, the untimely repair of these lesions may ultimately result in G:C to A:T transversion during replication, a mutagenic feature common in many cancers (3,4). While rapid removal of oxidized nucleotides is normally achieved by the base excision repair (BER) pathway (5), accumulation of oxidative DNA lesions is a feature of many conditions including cancer, Alzheimer's disease and Type II diabetes mellitus (6).

In human cells, 8-oxoG adducts are recognized and primarily removed by human 8-oxoguanine glycosylase 1 (hOGG1). hOGG1 is a bifunctional glycosylase that exhibits both DNA glycosylase and apurinic/apyrimidinic (AP) nuclease activities (7–10). Removal of the 8-oxoG base by hOGG1 leaves an abasic site, which can then be cleaved at the 3′-side via a β-elimination reaction. As hOGG1 has limited AP lyase activity, apurinic/apyrimidinic endonuclease 1 (APE1) may also catalyze cleavage of the AP site. APE1 functions to displace hOGG1 from the abasic site (11,12) and then nicks the phosphodiester backbone 5′ to the abasic residue, creating a free 3′-hydroxyl site. Gap filling and subsequent removal of the 5′-terminal deoxyribose phosphate residue is further catalyzed by DNA polymerase beta (POLB)­­, followed by DNA ligase III mediated closure of the phosphodiester backbone (13). As well as repairing the damaged nucleotide, the cell initiates a response that includes activation of the ataxia telangiectasia mutated (ATM) kinase and p53 (14).

Single-stranded DNA-binding (SSB) proteins are involved in virtually all DNA processing events, including replication, transcription and DNA repair (15,16). When DNA is damaged, SSB proteins detect the damage, protect the generated single-stranded DNA (ssDNA) and orchestrate a repair through the recruitment of other repair proteins (15,17). The SSB protein family is characterized by a structural Oligonucleotide/Oligosaccharide-binding fold (OB-fold) that binds ssDNA (18) and includes prokaryotic SSB, found in archaea and bacteria, as well as human proteins, such as RPA, hSSB1 and hSSB2. Interestingly, hSSB1 is closely related to the *Sulfolobus solfataricus* SSB and shares few similarities with *E. coli* SSB or human replication protein A (RPA). hSSB1 is considered a simple SSB, as each polypeptide contains only one OB-fold, while the more complex RPA contains multiple OB folds over a number of polypeptides (19).

hSSB1 has a critical function in the repair of double-strand DNA-breaks (DSBs) by homologous recombination (HR) (17,20–24). Following the induction of DSBs, hSSB1 rapidly localizes to the break site in a PAR-dependent manner (25). hSSB1 then functions to recruit the Mre11-Rad50-Nbs1 (MRN) complex, allowing activation of the ATM kinase and cell cycle checkpoints (20,21,24). hSSB1 also stimulates the nuclease activity of Mre11 to promote resection of the 5' DSB strand. hSSB1 may also function later in HR during strand invasion. In addition, we have demonstrated that hSSB1 is essential for the restart, signaling and repair of stalled replication forks (26).

Here, we establish a novel role for hSSB1 in the base excision repair pathway where it is required for cell survival following a oxidative stress. In the absence of hSSB1, human 8-oxoguanine glycosylase 1 does not localize to chromatin, resulting in the accumulation of 8-oxoguanine in the genome.

## EXPERIMENTAL PROCEDURES

### Cell lines and cell treatments

HeLa cells were maintained in Dulbecco's Modified Eagle medium (DMEM, Gibco) and U2OS cells were maintained in Roswell Park Memorial Institute medium (RPMI, Sigma). All cell culture media was supplemented with 10% fetal bovine serum (Sigma). For oxidative stress experiments, cells were cultured in a humidified atmosphere with 8% oxygen and 5% CO_2_ at 37°C. For experiments with cells exposed to ionizing radiation, cells were grown in an atmosphere of 21% oxygen and 5% CO_2_ at 37°C. To induce oxidative DNA damage, cells were treated with 250 μM of H_2_O_2_ or 30 mM potassium bromate (KBrO_3_), for 30 min in serum-free media. Media containing H_2_O_2_ or KBrO_3_ was removed and cells were washed multiple times with Phosphate-buffered saline (PBS) before incubation for the appropriate time in media containing serum.

### Expression constructs, siRNA and transfections

The mammalian expression vector containing the hSSB1 CDS (pCMV6-AN-3DDK) was supplied by Origene. Site-directed mutagenesis (SDM) was used to introduce the non-coding mutations for small interfering RNA (siRNA) resistance and was performed using the polymerase *Pfu* Ultra (Stratagene). The pET28a hSSB1 vector has been described previously. For the preparation of truncation mutants, premature STOP codons were introduced by SDM as per above. The preparation of hOGG1 point mutants has been performed on pGEX-hOGG1 vector, as described earlier. Primer sequences are listed in Supplementary Table S1.

Mammalian expression vectors were transfected using Lipofectamine 2000 (Life Technologies).

Stealth siRNA against hSSB1 were synthesized by Life technologies (Invitrogen). Individual siRNA sequences were (sense) 5′-GACAAAGGACGGGCAUGAGdTdT and (antisense) 5′-CUCAUGCCCGUCCUUUGUCdTdT (17). hOGG1 was targeted using either pooled esiRNAs (Sigma Aldrich) or the Silencer Select siRNA sequences (sense) 5′-GAUCAAGUAUGGACACUGAtt and (antisense) 5′-UCAGUGUCCAUACUUGAUCcg (Life Technologies). siRNAs were transfected using RNAiMax (Life Technologies).

### Antibodies

Cell Signaling Technology supplied all antibodies used in this study with the exception of anti-FLAG (Sigma), hOGG1 (Sigma) and 8-oxoG (Trevigen). Sheep antiserum against hSSB1 has been described previously (17). Control IgGs were from Sigma. Secondary antibodies used for immunoblotting were from LiCor, while secondary antibodies used for immunofluorescence were from Life Technologies (Invitrogen).

### Clonogenic survival assays

For siRNA experiments, U2OS cells were transfected with control siRNA, hSSB1 siRNA or hOGG1 siRNA and two days following siRNA transfection, 400 cells were seeded into 6 cm dishes. Cells were treated with various concentrations of H_2_O_2_ or KBrO_3_ for 30 min in serum-free medium. Following 10 days of culture, cells were fixed and stained with 4% methylene blue in methanol and colonies were counted manually. Assays were performed at least three times. Results are displayed as mean ± S.D. and significance was examined using a Student's *t* test with a *P* value of <0.05 considered significant.

### Neutral comet assay

Cells were lifted immediately following mock, H_2_O_2_, KBrO_3_ or ionizing radiation treatment and 10^3^ cells were mixed with 0.6% low-melting point agarose (Biorad) (37°C in 1 X TBE). The cell suspension was spread onto a comet slide (TREVIGEN) and immersed in lysis buffer (2.5 M NaCl, 100 mM EDTA, 10 mM Tris (pH10), 1% Triton X-100) for 30 min at 4°C. Slides were immersed in TBE for 15 min before electrophoresis at 70 volts (∼90 mA) for 30 min. Slides were then washed in dH_2_O and immersed in 70% ethanol for 5 min and dried at 45°C for 15–30 min. The DNA was stained using SYBR® Green I (Sigma) (1:10 000) before being dried completely and visualized using a Nikon Eclipse Ti microscope. Quantitation of comet tail moments was performed on a minimum of 50 cells using Image J plugin where the densitometry of the head and tail, as well as length are measured to calculate the comet tail moment. Assays were performed using at least three biological repeats. Results are displayed as mean ± S.D.

### Immunoprecipitation

To detect an association between hSSB1 with hOGG1, immunoprecipitation from U2OS cell lysates were performed as previously described (20). Protein concentration was determined by bicinchoninic acid (BCA) assay (Sigma). Equal amounts of lysate were incubated with the appropriate antibody or isotype IgG overnight at 4°C. Antibodies were captured using magnetic protein A/G Dynabeads (Invitrogen), washed five times in NP40 buffer (20mM HEPES, pH 8.0, 150 mM KCl, 10 mM MgCl2, 0.5 mM EDTA, 0.2% NP40, 5% glycerol) containing protease inhibitor cocktail (Sigma) and phosphatase inhibitors (Cell Signaling Technology) prior to immunoblot analysis.

### Immunofluorescence microscopy

Immunofluorescence was performed as described previously (17). Briefly, cells treated or mock-treated with H_2_O_2_ were grown on 96 well plates (IBIDI). Cells were pre-permeabilized in 20 mM HEPES (pH 8), 20 mM NaCl, 5 mM MgCl_2_, 1 mM ATP, 0.1 mM N_2_OV, 1 mM NaF and 0.5% NP40 for 15 min on ice. Following washes in PBS, cells were fixed in 4% paraformaldehyde for 20 min, washed again with PBS before blocking with 1% donkey serum in PBS for 30 min. Cells were then incubated with the appropriate antibody (hSSB1–1:300; hOGG1–1:200) in 0.5% donkey serum overnight in a humidified chamber at 4°C. Wells were washed with PBS and incubated with secondary antibodies for 45 min at ambient temperature before the nuclei were counterstained with 4′, 6-diamidino-2-phenylindole hydrochloride (DAPI; Sigma).

Images were collected on a Deltavision PDV microscope and collated for figures with Adobe Photoshop. Staining of 8-oxoG oxidative lesions was performed using an anti-8-oxoG antibody as per the supplier's instructions (Trevigen). Images were taken on a Nikon Eclipse Ti microscope. Cellprofiler software (Broad institute) was used for quantification, a mask was created using the DAPI channel to identify the nuclei, and then the total intensity of the AlexaFluor 488 channel was measured for each cell nucleus. A minimum of 1000 nuclei was quantified for each condition. Where relevant, results are displayed graphically as mean ± S.D. and analyzed using a student's *t* test with a *P* value of <0.05 considered significant.

### Slot blot

To examine 8-oxoG lesions after H_2_O_2_ treatment, U2OS cells were lysed in buffer (100 mM Tris, pH 7.6, 200 mM NaCl, 0.5 mM EDTA, pH 8.0, 0.5% SDS (w/v), 0.5% Triton X-100 (v/v), 0.2 mg/mL Proteinase K, 0.1 mM Deferoxamine mesylate salt (Sigma)) containing RNase A (Roche) and incubated at 55°C overnight. Chromosomal DNA was precipitated with isopropanol and re-suspended in TE buffer. DNA concentration was determined using a Nanodrop 2000 (Thermofisher). Equal amounts of DNA were immobilized on a nylon membrane using a 48-well Slot blot vacuum manifold. DNA was cross-linked onto the membrane using a UV Stratalinker before blocking with 2% v/v fish gelatin in PBS-T (0.05% Tween 20). Membranes were probed with the 8-oxoG antibody, washed with PBS-T and probed with an appropriate secondary antibody. Membranes were scanned on an Odyssey infrared imaging system (LiCor). Signal intensity was assessed by densitometry using the Odyssey software (LiCor). Results are displayed graphically as mean ± S.D. and significance was examined using a Student's *t* test with a *P* value of <0.05 considered significant.

### Subcellular protein fractionation

Cellular fractionation was performed using a subcellular fractionation kit for cultured cells (Life Technologies), as per the manufacturer's instructions. The protein concentration of each fraction was assessed by BCA assay. Equal amounts of each fraction were analyzed by immunoblot. Fraction purity was assessed by anti-actin and anti-histone H3 immunoblot analysis.

### Immunoblot

Immunoblots were performed as described previously (26) and visualized using an Odyssey infrared imaging system. When necessary, immunoblots were quantified using ImageJ software and normalized to actin.

### Quantitative real time PCR (qRT-PCR)

qRT-PCR reactions were performed in 384 well plates (Axygen) and contained 1 μl diluted cDNA reverse transcribed from total RNA (equivalent to 0.5 μg) in nuclease-free H_2_O (1:5), 50 nM forward and reverse primer, 1x final concentration of SYBR green PCR master mix (Applied Biosystems) and nuclease-free H2O (total volume of 10 μl). Reactions were performed using a ViiA7 real-time PCR system (Life Technologies). Cycling conditions were 95°C for 10 min, 40 cycles of 95°C for 15 s and 60°C for 1 min followed by a primer-template dissociation step. Gene expression was normalized to 7SL mRNA levels using the comparative CT (CT) method. The primer sequences for the human genes are in Supplemental Table S1.

### Protein purification

hSSB1 proteins harboring a N-terminal (His)_6_-tag was expressed in *E. coli* T7 shuffle strain (*Δgor ΔtrxB*) and purified as previously described (17). GST-OGG1 wild type and mutants were prepared as described previously (27). Both proteins were concentrated and resolved on a *Superdex 200 10/300* GL size exclusion chromatography column (GE healthcare), run with K buffer containing 300 mM KCl. Fractions containing proteins to near homogeneity were pooled, concentrated and stored at -80°C. GST was purified using an empty pGEX plasmid. Protein concentrations were estimated by running a dilution series on SDS-PAGE gel.

### Pull-down assays

Recombinant full length and truncated hSSB1 were immobilized on Cyanogen bromide-activated-Sepharose^®^ 4B (Sigma-Aldrich) according to the manufacturer's instructions. Incubation of proteins, washes and elution were performed as previously described (28).

### Electrophoretic DNA mobility shift assay

Unless otherwise stated, increasing concentrations of hSSB1 (0, 0.05, 0.1, 0.25, 0.5, 0.75, 1, 2 μM) were incubated with 90 fmol of synthetic substrates, in 10 μl of binding buffer (50 mM Tris-HCl pH 7.5, 100 mM KCl, 100 μg/ml BSA) for 15 min at 37°C. Samples were electrophoresed on 8% polyacrylamide gels and visualized using a Starion FLA-9000 image scanner (Fujifilm). Free DNA was quantified using MultiGauge software (Fujifilm), and bound DNA was plotted as the difference between a given free DNA and the total DNA without protein.

### Targeting of hOGG1 to DNA

Biotinylated dsDNA containing an 8oxoG lesion was immobilized on Dynabeads^®^ M-280 Streptavidin. 2 μg of recombinant hSSB1 and hOGG1 F319A were incubated together in 20 μl of buffer K with 75 mM KCl at 4°C prior being mixed with 5 μl of beads. After 2 h at 4°C, the supernatant was collected, beads were washed with K buffer containing 100 mM KCl and proteins were eluted from beads. Supernatant (S), Wwash (W) and Elution (E) were visualized on SDS-page gel stained with coomassie blue.

### Incision assays

Incision assays were performed in 20 μl as previously described (29) using 90 fmol of dsDNA substrate containing an 8-oxoG/C lesion at the 11th nucleotide position. 20 nM of recombinant hOGG1 and the indicated concentration of purified hSSB1 at 37°C for 30 min. The reactions were stopped using formamide loading dye with 100 mM NaOH and heated for 30 min at 95°C to promote complete strand cleavage of the abasic sites. Reactions were resolved on 20% polyacrylamide-7 mM Urea gels. Gels were scanned using Starion FLA-9000 image scanner and quantified using MultiGauge software.

## RESULTS

### hSSB1 relocalizes to chromatin after oxidative stress

**Figure 1.**
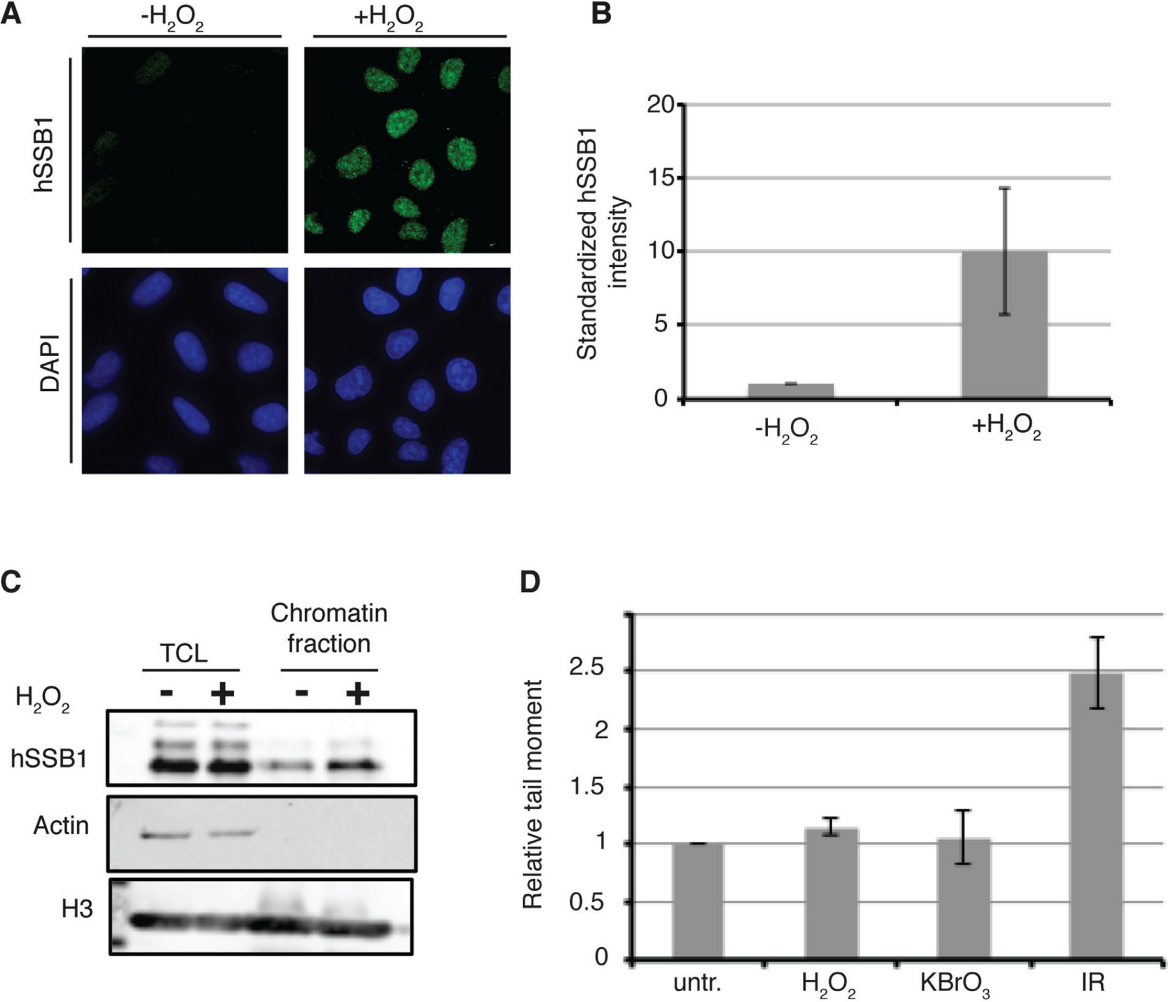
hSSB1 localizes to chromatin following oxidative stress. (**A**) Representative immunofluorescent staining of hSSB1 (green) and DAPI (blue) in pre-permeabilized, detergent washed and fixed U2OS cells cultured at 8% oxygen and treated with 250 μM H_2_O_2_ for 30 min, or left untreated. (**B**) Quantification of hSSB1 staining shown in Figure 1A. Total intensity of hSSB1 staining was measured for each cell nucleus. A minimum of 1000 nuclei were quantified. The intensity is standardized against the intensity measured in the untreated cells, and is given in arbitrary units. (**C**) hSSB1 localizes to chromatin after H_2_O_2_ treatment. U2OS cells cultured at 8% O_2_ were treated with 250 μM H_2_O_2_ for 30 min or left untreated. Cells were washed, collected and chromatin fractions were isolated as described in Experimental Procedures. Immunoblotting experiments were performed using indicated antibodies. TCL: total cell lysate. The image is representatives of at least 3 independent experiments. (**D**) Gross double strand breaks are not induced in cells treated with 250 μM H_2_O_2_, or 30 mM KBrO_3_. Alkaline comet assay was performed immediately after treatment. The comet tail moment is standardized against the tail moment measured in the untreated cells, and is given in arbitrary units. Data are graphed as mean ± SD from three independent experiments.

To explore the possibility that hSSB1 may function in the repair of oxidative DNA damage, we investigated whether hSSB1 is recruited to chromatin after oxidative stress. U2OS cells were cultured at 8% oxygen and treated with 250 μM H_2_O_2_ for 30 min to stimulate formation of oxidative DNA lesions. Following treatment, non-chromatin bound proteins were removed from the cell by detergent-based extraction and the cells were fixed prior to immunostaining with a hSSB1 antibody (17,30). When compared with non-treated cells, a ∼5-fold increase in hSSB1 staining at the detergent resistant chromatin was observed in the H_2_O_2_ treated cells, suggesting that hSSB1 relocalizes following oxidative stress (Figure 1A,B). To confirm this observation, immunoblots were performed on whole cell lysates and chromatin fractions prepared from U2OS cells following the same treatment. Membranes were probed using an anti hSSB1 antibody, this also demonstrated an increase in chromatin localization of hSSB1 in treated versus control cells (Figure 1C).

Although H_2_O_2_ treatment results in the generation of oxidized DNA bases within the genome, prolonged treatment is also associated with the formation of double-strand DNA breaks (31). As hSSB1 is loaded onto the chromatin following induction of DSBs(17), we tested whether the treatment conditions generated DSBs. To assess this, we used neutral comet assay to detect the formation of DSBs. When compared to untreated cells, cells exposed to H_2_O_2_ or potassium bromate, also known for inducing oxidative DNA damages, did not have a significantly increased tail length (Figure 1D, Supplementary Figure S1). As expected, tail moment was however increased following an exposure to 6 Gy irradiation. These findings suggest that chromatin localization of hSSB1 in response to H_2_O_2_ and KBrO_3_ treatments, is likely in response to oxidative damage to the genome.

### hSSB1 enables efficient ATM activation

**Figure 2.**
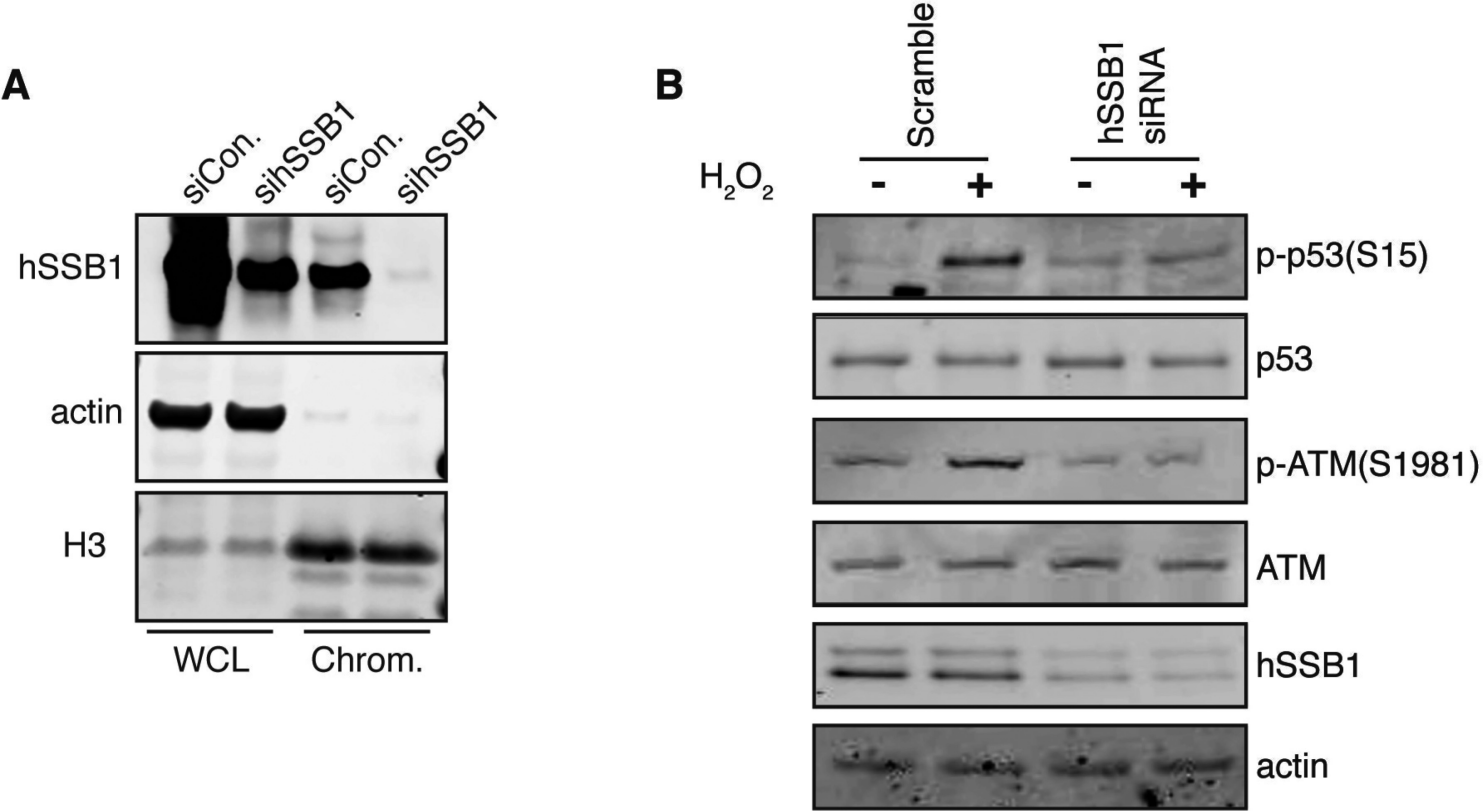
hSSB1 enables efficient ATM activation following oxidative stress. (**A**) hSSB1 depletion by siRNA. Whole cells lysate (WCL) and chromatin fraction (Chrom.) were collected from U2OS cells, following siRNA targeting using hSSB1 or siRNA control. Immunoblotting experiments were performed using indicated antibody. The immunoblot shown is representative of at least three independent experiments. (**B**) ATM signaling is not phosphorylated in hSSB1-depleted cells. Immunoblots of lysates from U2OS cells (Scramble) and hSSB1-depleted cells (hSSB1 siRNA), non-treated or treated with 250 μM H_2_O_2_ for 30 min, were probed for p53, phospho-p53, ATM, phospho-ATM, hSSB1 and β-actin antibodies. The images are representative of 3 independent experiments.

After oxidative stress, the ATM kinase is activated in a process that is marked by its auto-phosphorylation of serine 1981 (S1981) and by the stabilization of the ATM dimer by disulfide bridges (14). The ATM kinase subsequently phosphorylates p53 on Serine 15, resulting in p53 activation (32). hSSB1 is critical for activation of the ATM/ATR signaling response following DSB induction and is required for ATM phosphorylation (17). We therefore examined whether hSSB1 could have a similar role in ATM activation after the exposure of U2OS cells to oxidative stress. For this, U2OS cells were depleted of hSSB1 using a siRNA approach. Importantly, while total cellular levels of hSSB1 were reduced following transfection, minimal residual hSSB1 was also detected in the chromatin fraction (Figure 2A). Whole cell lysates were prepared from control or hSSB1-depleted U2OS cells following H_2_O_2_ or mock treatment and analyzed by immunoblot. Activation of ATM (S1981 phosphorylation) and p53 (S15 phosphorylation) occurred normally following oxidative stress in the control siRNA treated cells (scramble) and appears comparable to ATM activation following double strand break induction (Supplementary Figure S2 A), as previously described (14,33). Cells depleted of hSSB1 (hSSB1 siRNA) exhibited impaired activation of both ATM and p53 (Figure 2B).

### hSSB1 is required for the timely removal of 8-oxoguanine adducts and participates in cell survival

**Figure 3.**
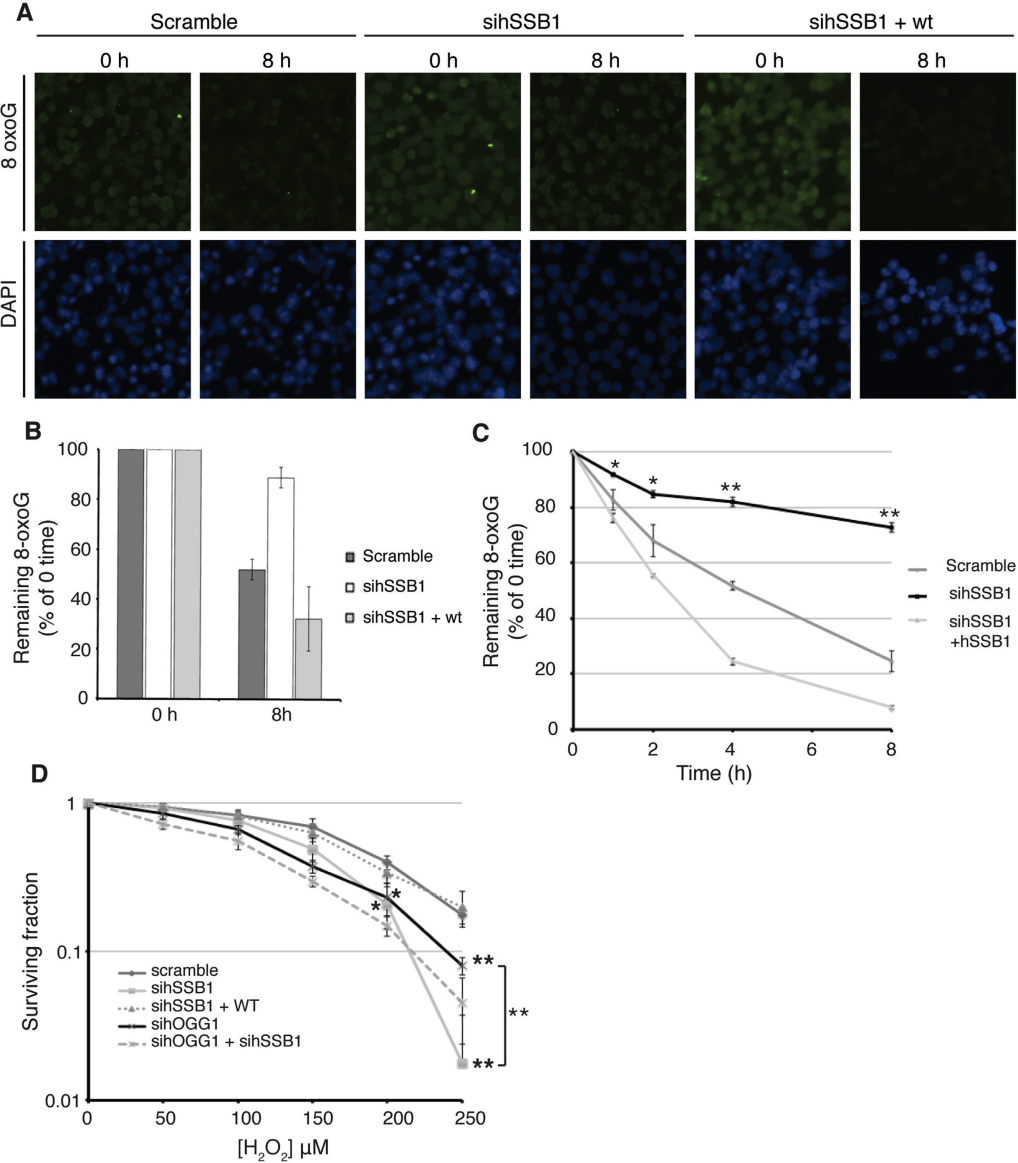
hSSB1 is required for the removal of 8-oxoG lesions. (**A**) 8-oxoGs persist in the absence of hSSB1. Immunofluorescence of 8-oxoGs (green) and DAPI (blue) of pre-permeabilized, detergent washed and fixed hSSB1-depleted U2OS cells, immediately after being treated with 250 μM H_2_O_2_ for 30 min, or an 8h recovery post treatment. (**B**) Quantification of the 8oxoG staining shown in Figure 3A. Total intensity of 8-oxoG staining was measured for each cell nucleus. A minimum of 1000 nuclei were quantified, (**: *P*<0.005). (**C**) Quantification of slot blot of the remaining 8-oxoGs from U2OS shown in Figure 3C. (see also Supplemental Figure S1). Data is graphed as mean ± SD from three independent experiments. (Scramble *vs*. sihSBB1; *: *P*<0.05, **: *P*<0.005). (**D**) Survival curve from a clonogenic assay of U2OS cells depleted for hSSB1, hOGG1 or both, treated with H_2_O_2_. Non-depleting negative control (scramble), sihSSB1, sihOGG1, sihSSB1 +siHOGG1 or sihSSB1 and a siRNA -resistant flag-tagged hSSB1 (+hSSB1) were transfected into cells. Cells were treated for 30 min with the indicated H_2_O_2_ concentration in serum free media, washed with PBS and cultured for 10 days in media containing serum. All points represent the mean ± SD from three independent experiments. P values were calculated using a standard student's *t* test. (*: *P*<0.05, **: *P*<0.005).

We next explored the possibility that hSSB1 may be involved in the repair of oxidized DNA within the genome. For this, we sought to determine if hSSB1 may function in the repair of 8-oxoguanine (8-oxoG) adducts, the most common form of purine modification resulting from oxidative stress. Control or hSSB1-depleted U2OS cells were fixed and stained for 8-oxoG lesions (Trevigen (29)), either immediately post H_2_O_2_ treatment or following 8 h recovery in H_2_O_2_ free media. Intriguingly, while cells transfected with control siRNA repaired the majority of their damaged DNA within 8 h, hSSB1-depleted cells were significantly impaired in their ability to remove the 8-oxoG lesions (Figure 3A–B). To determine whether reintroduction of hSSB1 could rescue repair of these oxidative lesions, siRNA resistant hSSB1 was expressed in cells depleted of hSSB1 (Supplementary Figure S3A). Consistently, the expression of siRNA resistant hSSB1 restored the ability of the cell to remove 8-oxoG (Figure 3A–B).

Slot blot analysis further confirmed our immunofluorescence observations, demonstrating an impaired removal of 8-oxoG adducts from DNA in hSSB1-depleted U2OS cells following H_2_O_2_ treatment. These results were similarly rescued by exogenous expression of siRNA-resistant hSSB1 and resulted in increased removal of 8-oxoG (Figure 3C, Supplementary Figure S3B). As shown in Supplementary Figure S3A, the increased rate of removal may be due to the increased level of hSSB1 expression following reintroduction of exogenous siRNA-resistant hSSB1. Taken together, these data indicate that hSSB1 participates in the removal of 8-oxoguanine following oxidative stress.

Given that hSSB1 may be involved in the cell response following oxidative stress and in removal of 8-oxoG lesions, we next assessed whether hSSB1 was also required for cell survival. Clonogenic survival assays were performed using U2OS cells transfected with control (scramble) or hSSB1-targeting siRNA and treated with increasing doses of H_2_O_2_. As shown in Figure 3D, cells depleted of hSSB1 displayed significantly fewer surviving colonies following H_2_O_2_ treatment (250 μM) when compared with cells treated with a control scramble siRNA (*P*<0.0005), indicating an increased sensitivity to oxidative stress (Figure 3D). The expression of siRNA resistant hSSB1 rescued this sensitivity.

Given that hydrogen peroxide could induce a broad spectrum of oxidative damages to the cells, we measured the repair of 8-oxoG following treatment with KBrO_3_. Consistent with results obtained by immunofluorescence, after H2O2 treatment, removal of 8-oxoG lesions was impaired in hSSB1-depleted cells (Supplementary Figure S3C, S3D). In a similar manner, clonogenic survival assay were performed and demonstrated that cells depleted from hSSB1 displayed a lower survival rate following potassium bromate exposure (Supplementary Figure S3E).

Taken together, these data indicate that hSSB1 participates in the cellular response to oxidative DNA base damage and to cell survival following oxidative stress.

### hSSB1 is required for hOGG1 localization at the chromatin after oxidative stress

The hOGG1 DNA glycosylase is responsible for removing 8-oxoG lesions from the genome of the cell. Our data indicate that cells depleted of hSSB1 are unable to efficiently repair this type of lesion, suggesting that hSSB1 may function with hOGG1. To investigate this, we first performed a clonogenic survival assay with cells depleted for hOGG1 in cells either proficient or deficient for endogenous hSSB1 (Figure 3D). As expected, the survival of hOGG1-depleted cells is significantly impaired following H_2_O_2_ treatment (*P*<0.005). Interestingly, when compared to hOGG1-depleted cells, cells deficient for hSSB1 are more sensitive to H_2_O_2_, indicating that hSSB1 could also participate in other cellular responses to oxidative stress. Double knock down of hOGG1 and hSSB1 did not lead to a significantly different cell survival when compared to the single knock down of hSSB1.

**Figure 4.**
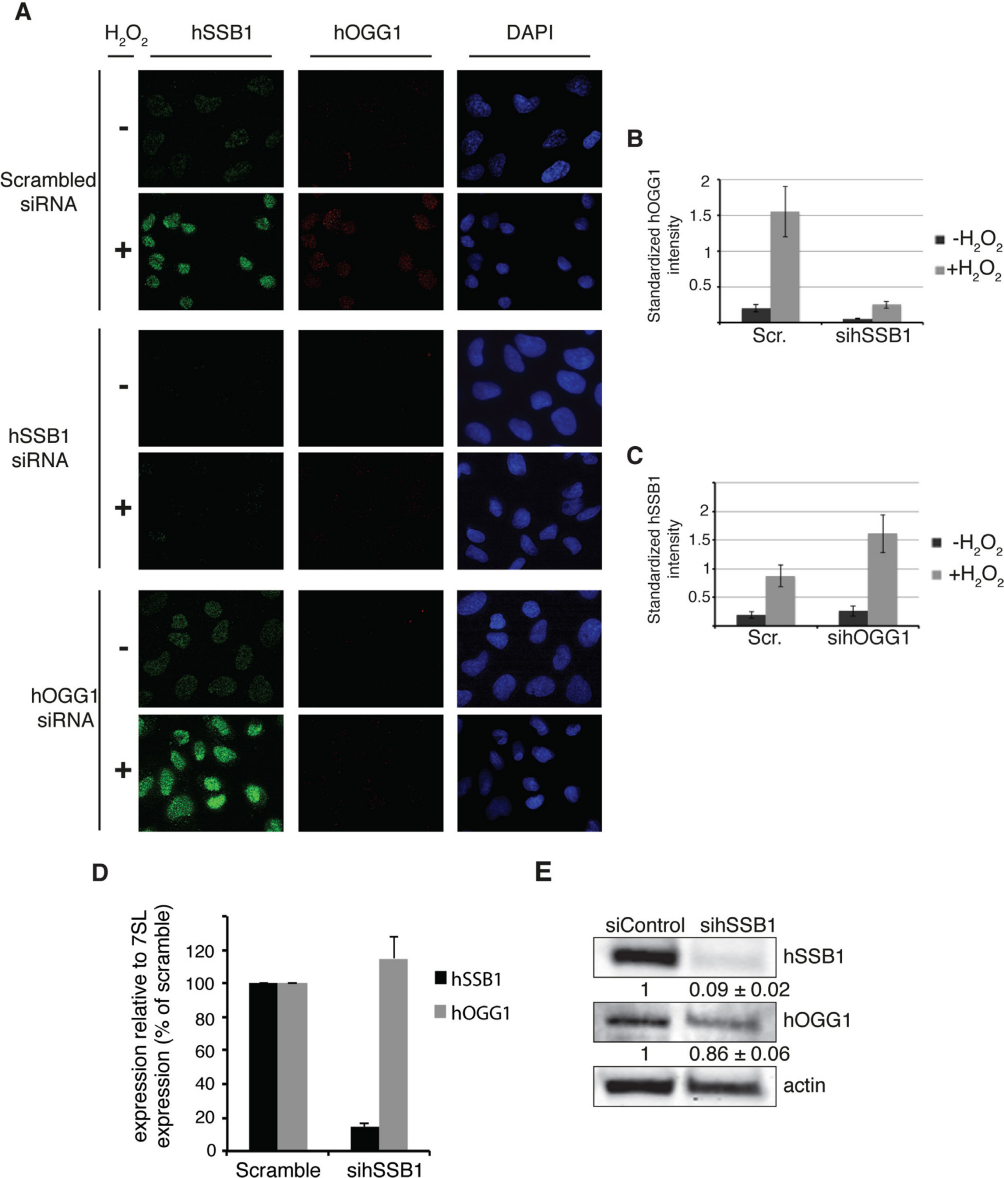
hSSB1 is necessary for hOGG1 localization to chromatin. (**A**) hOGG1 does not localize to chromatin in the absence of hSSB1. Immunofluorescence of hSSB1 (green), hOGG1 (red) and DAPI (blue) in pre-permeabilized, detergent washed and fixed hSSB1 or hOGG1-depleted U2OS cells cultured at 8% O_2_ and treated with or without 250 μM H_2_O_2_ for 30 min. (Scramble siRNA: Non-depleting control). (**B**) Quantification of hOGG1 staining shown in Figure 4A. Total intensity of hOGG1 staining was measured for each cell nucleus. A minimum of 1000 nuclei were quantified. (**C**) Quantification of hSSB1 staining shown in Figure 4A. Total intensity of hOGG1 staining was measured for each cell nucleus. A minimum of 1000 nuclei were quantified. (**D**) hOGG1 transcript levels are not altered by hSSB1 depletion. Real-time RT-PCR analysis of hSSB1 and hOGG1 mRNA expression in U2Os cells treated with or without siRNA targeting hSSB1. Bar graph representing the fold changes of mRNA levels quantified by normalization to the β-actin as an internal control. Mean values ± SD (n = 3). (**E**) hSSB1 depletion does not affect hOGG1 protein levels. Representative immunoblot analysis demonstrating the extent of hSSB1 depletion by siRNA in U2OS cells. Immunoblots were probed with anti-hSSB1, anti-OGG1 and anti-Actin antibodies. Numbers below each image are the levels of hSSB1 and hOGG1 relative to actin, represented as mean ± SD from three independent experiments (see also supplemental Supplementary Figure S3).

To explore the possible functional interplay between hSSB1 and hOGG1, we used immunofluorescence staining of hSSB1-depleted U2OS cells to monitor hOGG1 localization to chromatin following oxidative stress. Prior to fixation, cells were extracted with detergent to remove non-chromatin bound soluble proteins from within the nucleus, leaving only insoluble proteins bound to chromatin (17). While levels of both hSSB1 and hOGG1 were increased at the detergent-resistant chromatin ∼4.5- and ∼7-fold, respectively, following H_2_O_2_ treatment in scramble siRNA treated cells (Scramble, Figure 4A–C), minimal hOGG1 staining was observed in cells depleted of hSSB1 (Figure 4A–B). Furthermore, while the recruitment of hOGG1 to chromatin was dependent on hSSB1, hSSB1 loading to chromatin was not dependent on hOGG1 (Figure 4A, 4C). Importantly, increased levels of hSSB1 were seen to be recruited to the chromatin in cells depleted of hOOG1, which could reflect the accumulation of unrepaired 8-oxoG in these cells, as seen in Figure 3A–C. The absence of hOGG1 staining in hSSB1 knock down cells was not due to a loss of hOGG1 expression, as shown by qRT-PCR (Figure 4D). Furthermore, immunoblot analysis demonstrated only a marginal decrease in the hOGG1 polypeptide (<14% decrease in hOGG1) in hSSB1-depleted cells (Figure 4E). These data indicate a role for hSSB1 in the localization of hOGG1 to the chromatin following oxidative stress.

### hOGG1 and hSSB1 interact directly

**Figure 5.**
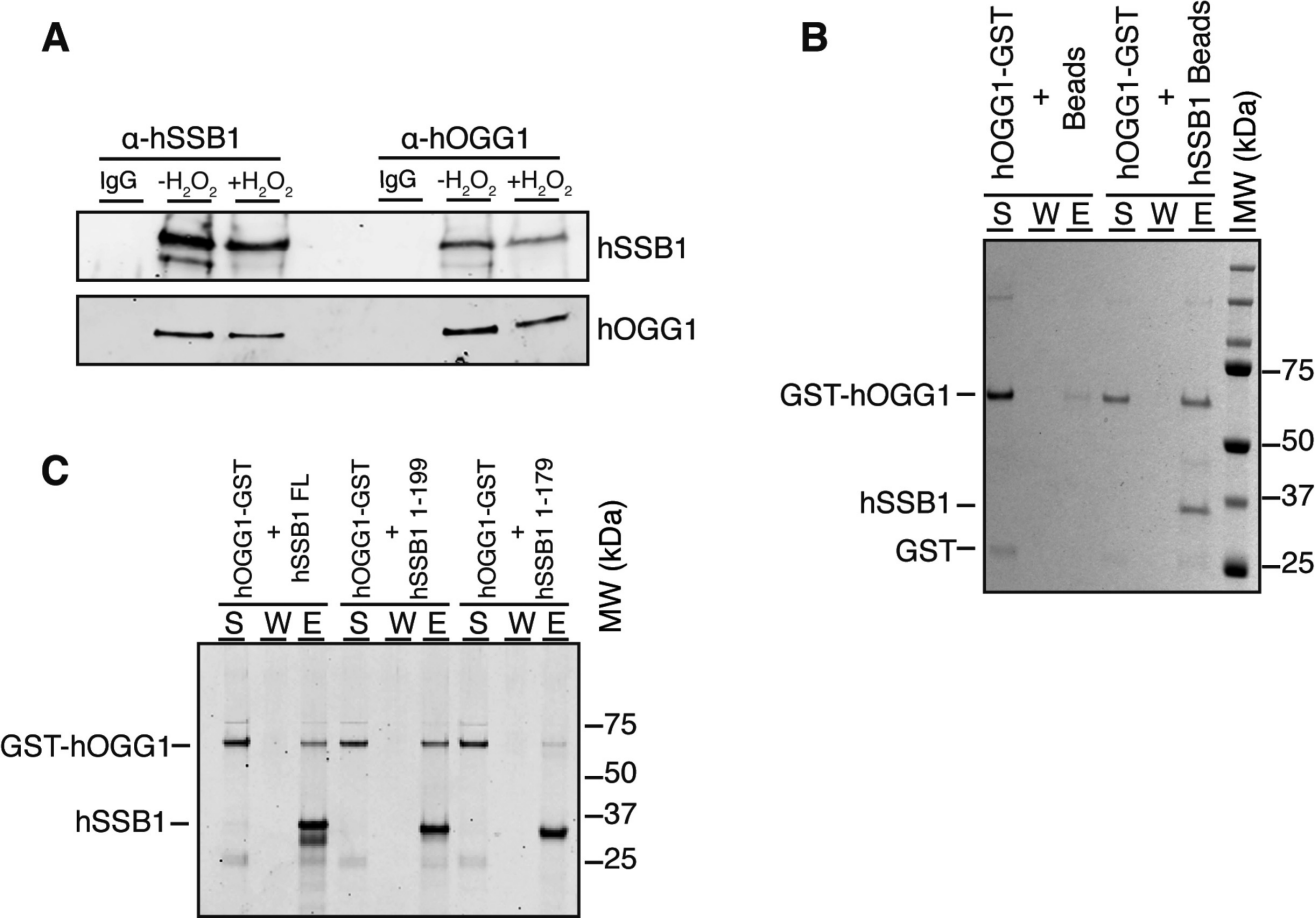
hSSB1 associates with hOGG1 to form a protein complex. (**A**) hOGG1 and hSSB1 form a complex independently of oxidative stress. Immunoprecipitation from U2OS cells treated or untreated with H_2_O_2_, using anti-hSSB1 or anti-hOGG1 antibodies were analyzed by immunoblot and probed with anti-hSSB1 and anti-hOGG1 antibodies. (**B**) hSSB1 interacts directly with hOGG1. 2 μg GST-tagged hOGG1 was incubated with hSSB1 immobilized on sepharose beads or beads alone. The beads were washed and treated with SDS to elute the bound proteins. The supernatant (S), wash (W), and SDS elute (E) were analyzed by SDS-PAGE and stained by Coomassie blue. (**C**) hSSB1 residues 179 to 199 mediate its interaction with hOGG1. 2μg GST-tagged hOGG1 was incubated with hSSB1 Full length (FL), hSSB1 1–199 or hSSB1 1–179 immobilized on sepharose beads. The beads were washed and treated with SDS to elute the bound proteins. The supernatant (S), wash (W), and SDS elute (E) were analyzed by SDS-PAGE and stained by Coomassie blue.

hSSB1 is known to form complexes with a number of other proteins, including the MRN complex and INTS3 (20,21,34). To explore if hSSB1 also forms a complex containing hOGG1, we performed reciprocal co-immunoprecipitation from U2OS cell lysates. As shown in Figure 5A, this indicated that hSSB1 and hOGG1 are likely to be in a complex together. To interrogate the possibility of a direct interaction between the two proteins, a series of pull-down assays were performed using purified recombinant hSSB1 and hOGG1. Purified recombinant hSSB1 was immobilized on sepharose beads and then incubated with purified GST-hOGG1. Following incubation, the supernatant was collected (S), the beads washed (W) and proteins bound to the beads were eluted (E). These results indicated that GST-hOGG1 interacts directly with hSSB1 (Figure 5B). Importantly, an interaction between hSSB1 and recombinant GST was not observed, while minimal background binding of hOGG1 to beads could be seen (Supplementary Figure S4). We next used truncated versions of hSSB1 to perform the same experiment and found hOGG1 to bind to a version of hSSB1 truncated of its last 20 amino acids (hSSB1 1–199) but not to a shorter hSSB1 fragment (hSSB1 1–179) (Figure 5C). This indicates that hSSB1 likely interacts with hOGG1 through the region between amino acids 179–199.

### hSSB1 binds to double-stranded DNA containing oxidative lesions

**Figure 6.**
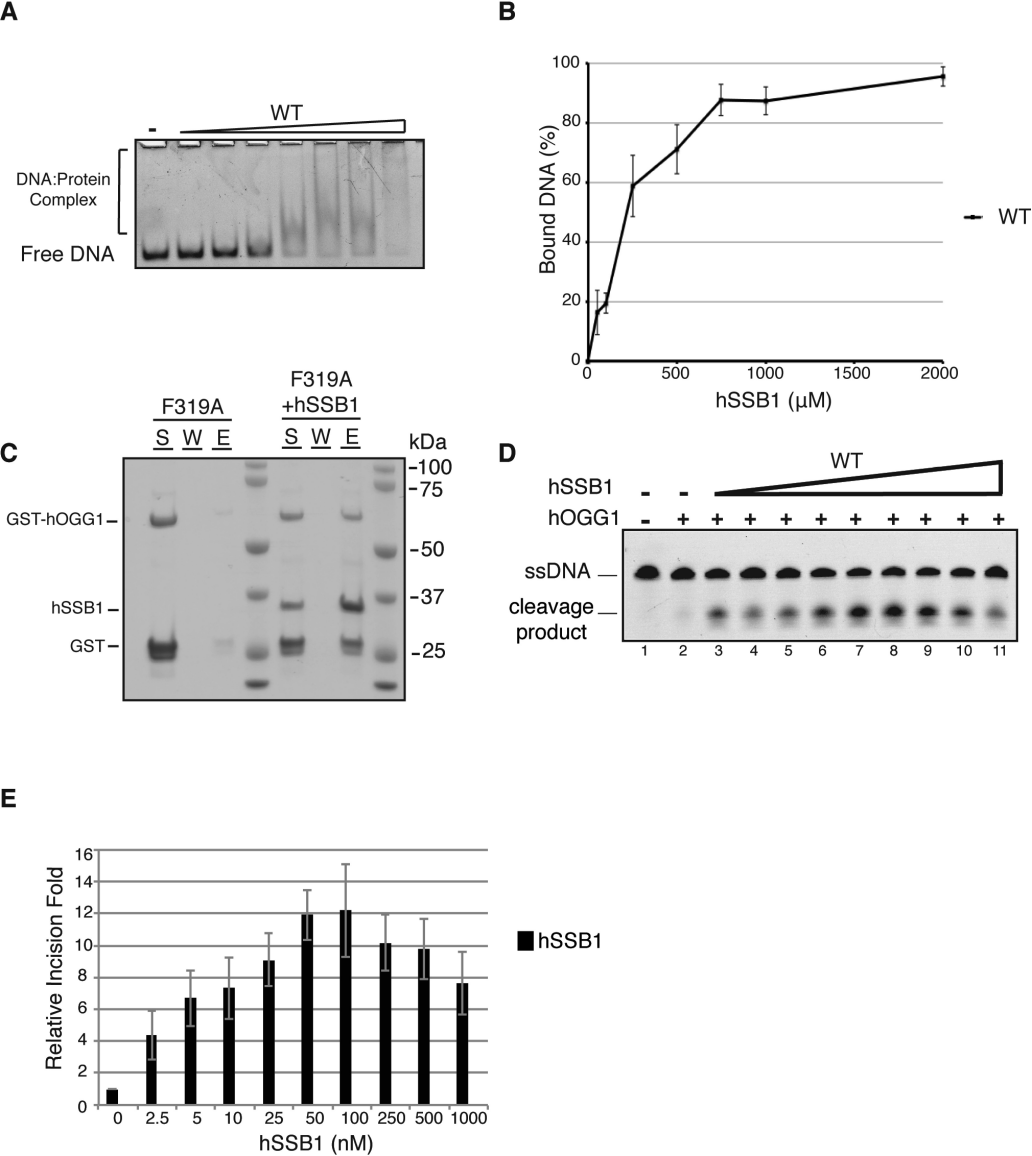
hSSB1 binds 8-oxoG containing dsDNA and enhances hOGG1 incision activity. (**A**) hSSB1 binds to 8-oxoGs containing dsDNA. Electromobility shift assay using 90 fmol of dsDNA containing a single 8-oxoG lesion, incubated at 37°C for 15 min, with increasing concentration (0, 0.05, 0.1, 0.25, 0.5, 0.75, 1, 2 μM) of hSSB1. (**B**) Quantification of A. Data graphed as mean ± SD from a minimum of 4 independent experiments. (**C**) hSSB1 promote hOGG1 recruitment to DNA containing a 8oxoG. hOGG1 F319A was incubated with dsDNA with a 8oxoG, immobilized on streptavidin beads, in the presence or absence of hSSB1. The beads were washed and treated with SDS to elute the bound proteins. The supernatant (S), wash (W), and SDS elute (E) were analyzed by SDS-PAGE and stained by Coomassie blue. (**D**) hSSB1 enhances hOGG1 incision activity. Representative gel of 8-oxoG cleavage reaction, carried in the presence of 20 nM of recombinant hOGG1 and indicated concentration of recombinant hSSB1. Reactions were stopped by addition of NaOH to cleave the abasic site generated, and resolved on an acrylamide/urea gel and visualized using a Starion scanner. (**E**) Quantification of D. Data graphed as mean ± SD from at least 3 independent experiments.

Our data indicate that hSSB1 relocalizes to chromatin following oxidative stress and is required to facilitate removal of 8-oxoG lesions. As hSSB1 is a DNA binding protein, we explored the possibility that hSSB1 could bind to double-stranded DNA (dsDNA) containing a single 8-oxoG. While hSSB1 did not bind to dsDNA in an electrophoretic mobility shift assay (Supplementary Figure S5), hSSB1 was able to bind duplexed DNA containing a single 8-oxoG as visualized by a shift in the migration of the DNA substrate (Figure 6A, B). These results indicate that hSSB1 can recognize and bind to duplex DNA containing a single 8-oxoG.

### hSSB1 recruits hOGG1 to dsDNA containing a 8-oxoG

Having demonstrated that hSSB1 forms a complex with hOGG1 and binds dsDNA with a 8oxoG, we next question whereas hSSB1 was promoting the recruitment of hOGG1 to the damaged DNA. For this, we used a hOGG1 mutant (F319A) that has been shown to be defective for 8oxoG recognition. Indeed, when incubated with biotinylated dsDNA containing a 8oxoG immobilized on streptavidin beads, F319A failed to be recruited to the DNA. However, when incubated with hSSB1, prior to being mixed the DNA, hOGG1 F319A was found in the fraction eluting from the beads (Figure 6C). This demonstrates that hSSB1 participates in the recruitment of hOGG1 to the damaged DNA.

### hSSB1 stimulates hOGG1 incision rate

We next examined if hSSB1 could enhance the activity of hOGG1 to remove 8-oxoG in an *in vitro* reconstituted assay. To explore this, a fluoroscently labeled 22 bp dsDNA substrate containing a single central 8-oxoG opposed to a cytosine was incubated with hOGG1 and increasing concentrations of hSSB1. Cleavage products resulting from 8-oxoG removal were analyzed on denaturing gels. While recombinant hSSB1 alone is unable to promote the cleavage of the substrate (Supplementary Figure S5C), hSSB1 stimulated the activity of hOGG1 by up to 12-fold in this assay (Figure 6D, E). At higher concentration, an inhibitory effect can be seen and is likely to be due to hOGG1 being depleted from its substrate by hSSB1. Interestingly, these data point out that 4–5 molecules of hSSB1 are associating with a single molecule of hOGG1. In addition, we also investigated whether hSSB1 alone or with increasing concentration of hOGG1 could promote the cleavage of dsDNA with a 8-oxoG matched to adenine. As expected, no cleavage activity was observed (Supplementary Figure S5D).

These data are consistent with hSSB1 being important for the recruitment and activity of hOGG1 at sites of oxidative damage.

## DISCUSSION

hSSB1 is known to play a crucial role in the repair of double-strand DNA-breaks by homologous recombination (17,20,21). Our data presented here now show that hSSB1 also plays an important role in the response to oxidative stress, with two separate functions, in ATM activation and in the removal of 8-oxoguanine by hOGG1-mediated base excision repair. Firstly, we demonstrated that hSSB1 localizes to chromatin and is necessary for cell survival following oxidative stress. We then show that hSSB1 is required for activation of the ATM kinase and its downstream target p53, as well as recruitment of the hOGG1 glycosylase to chromatin. In addition, we find that hSSB1 can stimulate the glycosylase activity of hOGG1 likely by increasing hOGG1 recruitment to DNA, promoting removal of the oxidized base and completion of BER.

Further to its role in the repair and signaling of double-strand DNA breaks during the process of homologous recombination, ATM is also activated following oxidative stress (14). Upon double-strand DNA breaks formation, ATM activation is observed by auto-phosphorylation on Serine 1981 and the disruption of the ATM dimer in an MRN and hSSB1-dependent manner (17,35). In contrast, following oxidative stress, the ATM kinase remains as a dimer, stabilized by disulfide bonds. Phosphorylation of ATM on Serine 1981 requires this dimerization, which is independent of MRN. Interestingly, we have now demonstrated that hSSB1 is required to facilitate ATM autophosphorylation following both DSB formation and oxidative stress. While ATM autophosphorylation is necessary for downstream p53 phosphorylation at serine 15 following DSBs formation, in the case of oxidative stress, ATM phosphorylation, is dispensable for p53 phosphorylation (14). In order to induce p53 phosphorylation following oxidative stress, ATM dimerisation is required. Interestingly, cells depleted of hSSB1 exhibit deficient p53 phosphorylation potentially indicating that ATM is not stabilized as a dimer in the absence of hSSB1. This would require further investigation to decipher this interplay.

ATM-dependent p53 phosphorylation may promote cell cycle arrest or apoptosis, however p53 may also have other functions following oxidative stress. For example, p53 transcriptionally activates the *OGG1* gene, which contains a p53 response element in the promoter (36). Our data suggest that this process is not dependent on p53 phosphorylation at serine 15, as hSSB1-depleted cells exhibit *OGG1* transcript levels comparable to the wild type cells. At the protein level, p53 has also been implicated in enhancing 8-oxoG removal by interacting directly with hOGG1 (37). Similarly, p53 also stimulates APE1 activity and stabilizes DNA polymerase beta at the abasic site created by the removal of the base adduct by the glycosylase (38). While the N-terminus region of p53, containing serine 15, appears to play a role in the stimulation of base excision repair, the exact role of the phosphorylation at serine 15 in this response is yet to be elucidated. As such, the necessity of serine 15 phosphorylation following oxidative stress could explain the decreased survival of hSSB1-depleted cells when compared to those depleted of hOGG1.

Previously we have shown that hSSB1 is recruited to chromatin following double strand break formation. Our current study demonstrates that following oxidative stress, hSSB1 is also loaded onto chromatin. While oxidative stress may also cause double-strand DNA breaks, we found under our experimental conditions that gross levels of DSBs were not detectable. However, we cannot discount the possibility that low levels of DSBs do form following H_2_O_2_ treatment. Moreover, H_2_O_2_ also induces the formation of single strand breaks. While we have not investigated if hSSB1 participates in single strand break repair, we cannot exclude that hSSB1 may also function in this process. Nonetheless, we demonstrate that hSSB1 is required for the removal of the most common oxidative DNA base damage within the cell, 8-oxoG. Herein we also demonstrate that hSSB1 forms a protein complex with the DNA glycosylase hOGG1 and that in the absence of hSSB1, hOGG1 cannot localize to chromatin.

hOGG1 is a bifunctional DNA glycosylase that can both hydrolyze the N-glycosylic bond between the target base and deoxyribose as well as cleave the phosphodiester bond 3′ to the AP site (AP lyase activity) (39). While the modality of binding to the 8-oxoG is well documented (8,40), the selective recognition of the damaged base versus unmodified guanine is still not well understood. A model has been proposed whereby hOGG1 scans along DNA, until it localizes with a 8-oxoG (41). The 8-oxoG lesion is flipped into the active site of hOGG1 where it binds more strongly than an unmodified guanine. However, this scanning does not explain why Amouroux *et al*., found that a F319A hOGG1 mutant that is unable to recognize 8-oxoG residues was still recruited to euchromatin following oxidative stress (42). The authors demonstrated that following oxidative stress the hOGG1 mutant was still able to localize to open chromatin regions and to promote assembly of the base excision repair centers, indicating that the re-localization of hOGG1 to oxidative lesions is independent of its ability to recognize and bind directly to such lesions (42,43). After oxidative damage, we observed that hOGG1 localized to the chromatin, an observation consistent with a previous study showing that hOGG1 redistributes from a nucleoplasmic fraction into nuclear foci following UVA treatments (44). However, in the absence of hSSB1, hOGG1 was not seen to localize to the chromatin, leading to the persistence of the 8-oxoG lesions in the genome. *In vitro* we demonstrate that hSSB1, while unable to bind to conventional dsDNA substrates, can bind to the same substrate containing a single 8-oxoG (substitution for a G). At the same time, hSSB1 physically interacts with hOGG1. Using the same mutant (F319A) published by Amouroux *et al*., we were able to demonstrate that hSSB1 promote the recruitment of hOGG1 to the substrate even in when hOGG1 cannot recognize the lesion. From our data, we can hypothesize that hSSB1 acts as an additional sensor of the lesion, guiding and anchoring hOGG1 to the damaged base, however we cannot rule out that hSSB1 also enhances hOGG1 enzymatic activity. These findings are consistent with the observations of Amouroux *et al*.

In light of the data presented here, we propose a new role for hSSB1 in the removal of 8-oxoguanine within the genome and the activation of ATM following oxidative stress. Understanding how hSSB1 localizes to the damaged base warrants further investigation as thermodynamic analysis of 8-oxoG containing DNA highlights the absence of any liberated ssDNA, (45) which was until this study the only known substrate for hSSB1 nucleation. Collectively our data suggest a novel role through which hSSB1 functions to protect the cell from oxidative damage, preventing mutations that may result in cancer and non-malignant diseases such as dementia and non-insulin dependent diabetes mellitus.

## Supplementary Material

SUPPLEMENTARY DATA
