# Supplementary material for: hSSB1 (NABP2/ OBFC2B) is required for the repair of 8-oxo-guanine by the hOGG1-mediated base excision repair pathway
Source: Nucleic Acids Res. 2015 Oct 10;43(18):8817–29. doi: 10.1093/nar/gkv790 (PMC4605301; doi:10.1093/nar/gkv790)
Supplement: SUPPLEMENTARY DATA [file supp_43_18_8817__index.html]

hSSB1 (NABP2/ OBFC2B) is required for the repair of 8-oxo-guanine by the hOGG1-mediated base excision repair pathway — hSSB1 (NABP2/ OBFC2B) is required for the repair of 8-oxo-guanine by the hOGG1-mediated base excision repair pathway — SUPPLEMENTARY DATA 

# hSSB1 (NABP2/ OBFC2B) is required for the repair of 8-oxo-guanine by the hOGG1-mediated base excision repair pathway

## SUPPLEMENTARY DATA

- SUPPLEMENTARY DATA
