# Supplementary material for: hSSB1 (NABP2/ OBFC2B) is required for the repair of 8-oxo-guanine by the hOGG1-mediated base excision repair pathway
Source: Nucleic Acids Res. 2015 Oct 10;43(18):8817–29. doi: 10.1093/nar/gkv790 (PMC4605301; doi:10.1093/nar/gkv790)

**­­­Supplemental figures legends**

**Supplemental figure S1:**

(A) Representative images of neutral comet assay experiments. The assay was performed on U2OS cells treated with 250 µM H_2_O_2_  or 30mM KBrO_3_ for 30min, or 6 Gy irradiation.

**Supplemental figure S2:**

**(A)** ATM activation after oxidative stress is comparable with the one after irradiation. Western blots from cell lysates of HeLa treated with or without H_2_O_2_ or with and without 6 Gy of ionizing radiation, were probed for ATM and phospho-ATM.

**Supplemental figure S3:**

(A) Over expression of the siRNA resistant hSSB1 construct. Western blot of U2OS cells non treated or treated with sihSSB1, and cell expressing exogenous siResistant hSSB1 wild type.

(B) Representative image of a slot blot from U2OS cells treated with control, sihSSB1 and sihSSB1 + siRNA resistant hSSB1, treated with 250 µM H_2_O_2_ for 30 minutes and allowed to recover for the indicated times. Whole cell lysates were probed with a specific anti 8-oxoG antibody. Quantification is shown in figure 2B and was obtain from at least 3 independent experiments.

(C) 8-oxoGs persist in the absence of hSSB1. Immunofluorescence of 8-oxoGs (green) and DAPI (blue) of pre-permeabilized, detergent washed and fixed hSSB1-depleted U2OS cells, immediately after being treated with 30mM KBrO_3_ for 30 min, or after an 8h recovery post treatment.

(D) Quantification of the 8oxoG staining shown in Figure S3C. Total intensity of 8-oxoG staining was measured for each cell nucleus. A minimum of 1000 nuclei were quantified, (**: p<0.005).

(E) Survival curve from a clonogenic assay of U2OS cells depleted for hSSB1, hOGG1 or both, treated with KBrO_3_. Non-depleting negative control (scramble), sihSSB1, sihOGG1, sihSSB1 +siHOGG1 or sihSSB1 and a siRNA -resistant flag-tagged hSSB1 (+hSSB1) were transfected into cells. Cells were treated for 30 min with the indicated KBrO_3_ concentration in serum free media, washed with PBS and cultured for 10 days in media containing serum. All points represent the mean ± SD from three independent experiments. P values were calculated using a standard student’s *t* test. (*: p<0.05, **: p<0.005).

**Supplemental figure S4:**

(A) Pull down assay controls using recombinant hSSB1 and GST-hOGG1. 2 μg GST was incubated with hSSB1 immobilized on sepharose beads, showing that hSSB1 does not interact with GST. 2 μg GST-hOGG1 was incubated with sepharose beads, revealing a background binding to the beads. The beads were washed and treated with SDS to elute the bound proteins. The supernatant (S), wash (W), and SDS elute (E) were analyzed by SDS-PAGE and stained by Coomassie blue

**Supplemental figure S5:**

(A) hSSB1 binding to dsDNA. Representative gels from an Electrophoretic Mobility Shift Assay: 90 fmol of 5’ FAM labeled dsDNA were incubated with increasing concentration of hSSB1 (0, 0.5, 1.0, 1.5, 2.0, 4.0 μM) at 37°C for 15 min. Reactions were resolved on 8% polyacrylamide gels and visualized using a Starion FLA-9000 image scanner.

(B) hSSB1 binding to dsDNA containing a single 8-oxoG. Representative gel from an Electrophoretic Mobility Shift Assay: 90 fmol of 5’ FAM labeled substrate were incubated with increasing concentration of hSSB1 (0, 0.1, 0.25, 0.5, 0.75, 1, 2 μM) at 37°C for 15 min. One reaction with 2 μM was treated with 1%SDS and 1mg/mL Proteinase K for 5min at 37°C. Reactions were resolved on 8% polyacrylamide gels and visualized using a Starion FLA-9000 image scanner.

(C) hSSB1 does not cleave dsDNA with a 8-oxoG, in the absence of hOGG1. Representative gel of 8-oxoG cleavage reaction, carried in the presence of 20 nM of recombinant hOGG1 and indicated concentration of recombinant hSSB1. Reactions were stopped by addition of NaOH to cleave the abasic site generated, and resolved on an acrylamide/urea gel and visualized using a Starion scanner.

(D) hSSB1 hSSB1 does not cleave dsDNA with a 8-oxoG matched to an adenine. Representative gel of 8-oxoG cleavage reaction, carried in the presence of 20 nM of recombinant hOGG1 and indicated concentration of recombinant hSSB1. Reactions were stopped by addition of NaOH to cleave the abasic site generated, and resolved on an acrylamide/urea gel and visualized using a Starion scanner.

**Supplementary Table 1:**

| **Oligo name** | **DNA sequence (5’ to3’)** |
| --- | --- |
| 8oxoG forward | CTCTCCCTTC-oxo-CTCCTTTCCTCT |
| Reverse C | AGAGGAAAGGAG**C**GAAGGGAGAG |
| Reverse A | AGAGGAAAGGAG**A**GAAGGGAGAG |
| siRNA resistant forward | cgagtgaccaagacaaaggatggacatgaggttcggacctgcaaag |
| siRNA resistant reverse | ctttgcaggtccgaacctcatgtccatcctttgtcttggtcactcg |
| hSSB1 forward | AGCCAAACCCAGAGTACAGC |
| hSSB1 reverse | CTGGTTCTCAGAGGCTGGAG |
| hOGG1 forward | CCGAGCCATCCTGGAAGAAC |
| hOGG1 reverse | CCATCAGGCAGATGCAGTCA |
| 7SL forward | ATCGGGTGTCCGCACTAAGTT |
| 7SL reverse | CAGCACGGGAGTTTTGACCT |
| V199STOP  forward | ggcccttccagcaacccttaaagtaacggcaaagaaacccgg |
| V199STOP  reverse | ccgggtttctttgccgttactttaagggttgctggaagggcc |
| I179STOP  forward | cccacccagcacccgatagactcgaagccagcc |
| I179STOP  reverse | ggctggcttcgagtctatcgggtgctgggtggg |
| OGG1 F319A forward | GCAGGTCGGCACTGGCCAGCACCGCTTGGG |
| OGG1 F319A reverse | cccaagcggtgctggccagtgccgacctgc |


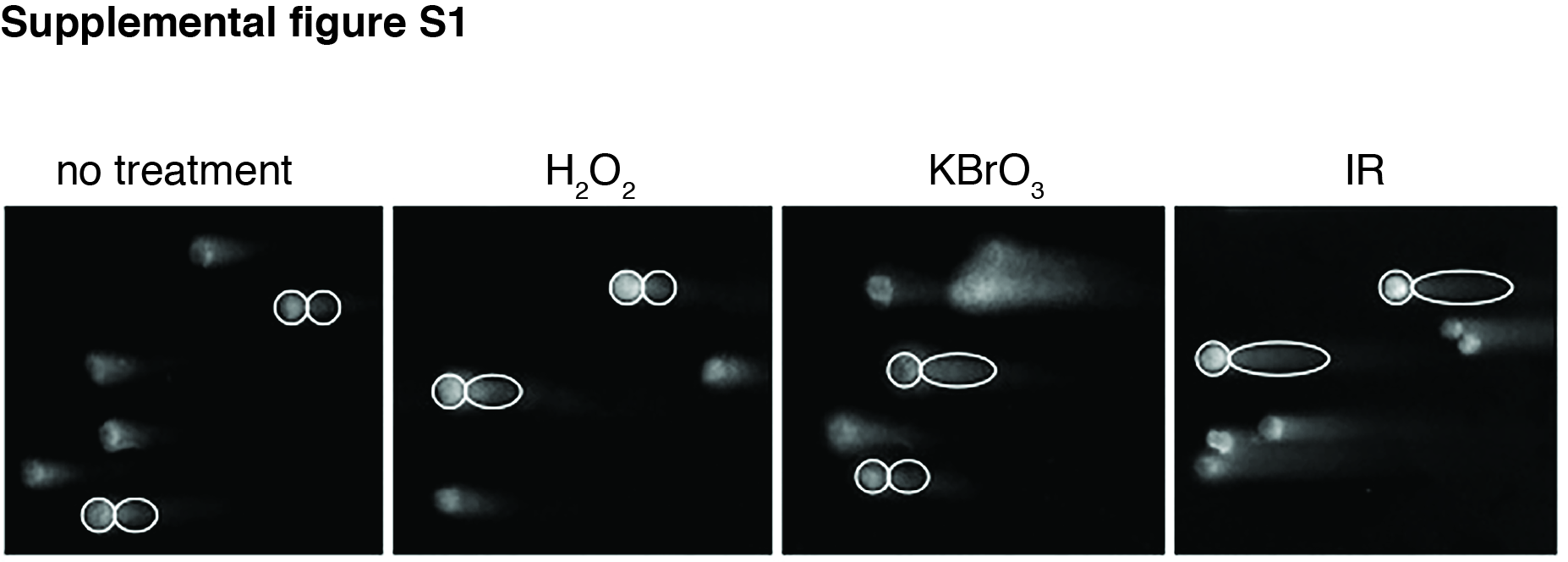


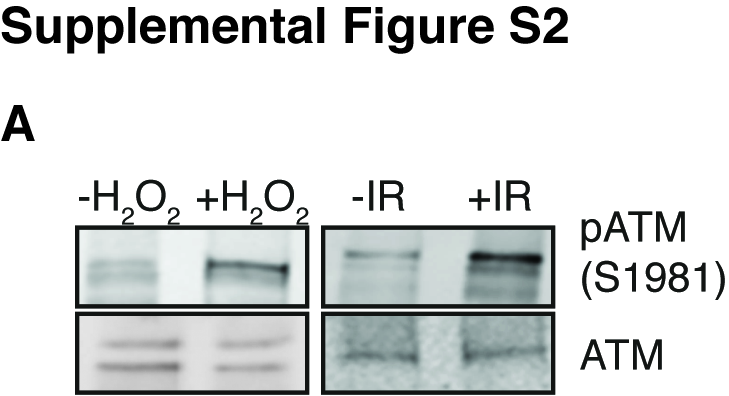


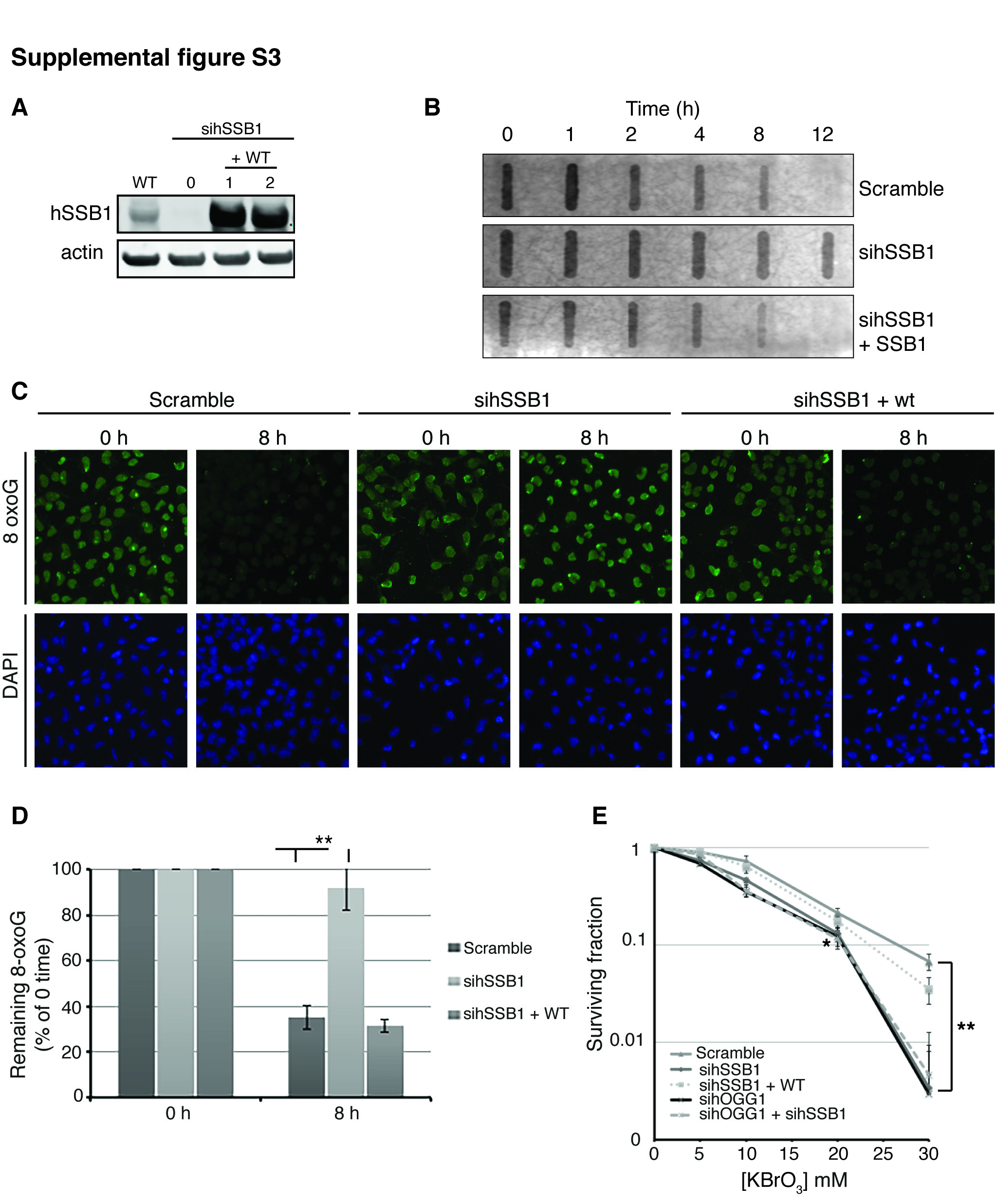


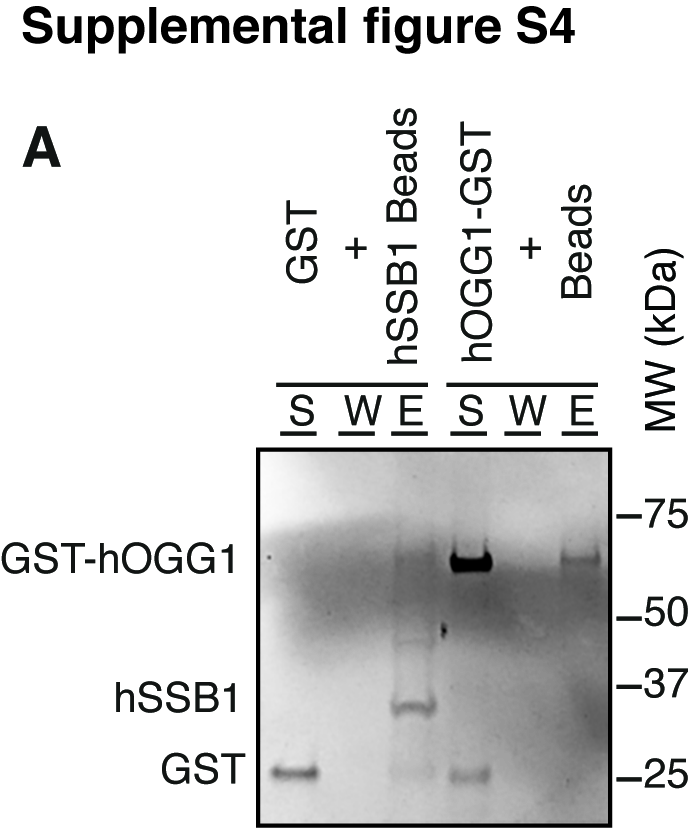


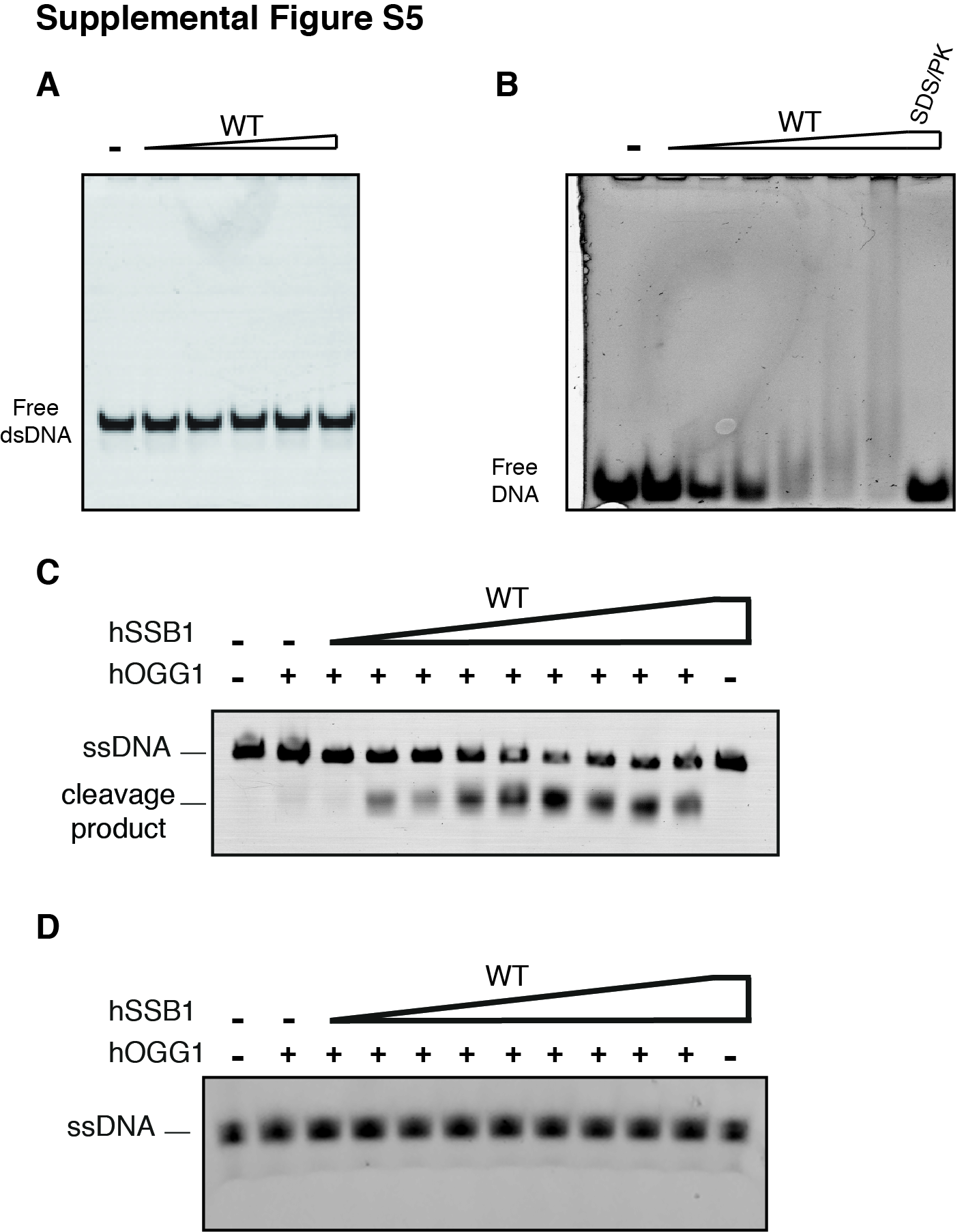

Supplement: SUPPLEMENTARY DATA [file supp_gkv790_nar-01348-d-2015-File008.docx]
